# *Plant Methods: *putting the spotlight on technological innovation in the plant sciences

**DOI:** 10.1186/1746-4811-1-1

**Published:** 2005-08-18

**Authors:** Brian G Forde, Michael R Roberts

**Affiliations:** 1 Editor-in-Chief, Plant Methods, Dept. of Biological Sciences, Lancaster University, Lancaster, LA1 4YQ, UK; 2 Deputy Editor, Plant Methods, Dept. of Biological Sciences, Lancaster University, Lancaster, LA1 4YQ, UK

## Abstract

*Plant Methods *is a new journal for plant biologists, specialising in the rapid publication of peer-reviewed articles with a focus on technological innovation in the plant sciences. The aim of *Plant Methods *is to stimulate the development and adoption of new and improved techniques and research tools in plant biology. We hope to promote more consistent standards in the plant sciences, and make readily accessible laboratory and computer-based research tools available to the whole community. This will be achieved by publishing Research articles, Methodology papers and Reviews using the BioMed Central Open Access publishing model. The journal is supported by a prestigious editorial board, whose members all recognise the importance of technological innovation as a driver for basic science.

## Editorial

Technical innovation is surely one of the most important catalysts for progress in any scientific discipline – and this must be particularly true for plant biology in the current post-genomics era. In launching *Plant Methods *at this time our principal goal is to give technical creativity in the plant sciences the high profile platform it needs and deserves. By providing a forum for the rapid publication of novel or significantly improved techniques in plant biology, *Plant Methods *will aim to stimulate the development and the adoption of new and improved techniques, tools and technologies in the plant sciences. By spotlighting the technical aspects of plant biology, and providing a focal point for discussion and exchange of information, we hope that *Plant Methods *will also be able to serve the plant community by promoting higher and more consistent standards in the practice of plant research.

The open access policy of *Plant Methods*, as described in the BioMed Central Open Access Charter [1], is one that has major advantages for authors and readers alike. For authors, Open Access ensures that their work is disseminated to the widest possible audience, given that there are no barriers to access. Since authors will hold the copyright they are also free to reproduce and distribute their own work (for example by placing it on their institution's website) and may grant to anyone the right to reproduce and disseminate the article, provided that it is correctly cited and no errors are introduced. It has been suggested that free online articles are more often viewed and more highly cited because of their easier availability [2]. For readers, the information available to them is not limited by their library's budget but is universally, freely and permanently accessible via the Internet. Consequently a country's economic status will not negatively influence its scientists' ability to access research. For these reasons we believe that the Open Access model represents the ideal format for achieving our objectives in launching *Plant Methods*.

Two main formats will be available for the publication of original work in *Plant Methods*: Research Papers, which will report a significant technical advance and its successful application to shed important new light on a biological problem, and Methodology Papers, which will describe an important new technique or research tool (such as a new online database or genetic stocks), but with less emphasis on the generation of new data beyond the required proof of concept. Too often researchers, particularly young researchers, are discouraged from dedicating the time needed for the development of new techniques or community resources because there is little opportunity to obtain proper recognition for their contribution. For example, it could easily take the major portion of a postdoctoral appointment to work through the cycles of trial and error needed to refine a new protocol, leaving little time to obtain the biological insights that a conventional research paper will demand. By providing a high profile outlet for methodology papers *Plant Methods *will encourage and reward technical creativity. Additional formats, including Reviews and Protocols, will be by invitation or by prior arrangement with a member of Editorial Board.

Our Editorial Board  brings together a group of internationally renowned plant scientists who share a strong belief in the fundamental importance of technological innovation. All manuscripts submitted to the journal will be rigorously peer-reviewed and papers will be published online immediately after acceptance and soon after listed in PubMed. When a paper has been accepted, an Article Processing Charge (APC) of £410 (approximately US$720) becomes payable. This pays for the article to be freely and universally accessible in various formats online, and for the processes required for inclusion in PubMed and archiving in PubMed Central. All *Plant Methods *articles will be archived in PubMed Central [3], the US National Library of Medicine's full-text repository of life science literature, and also in repositories at the University of Potsdam [4] in Germany, at INIST [5] in France and in e-Depot [6], the National Library of The Netherlands' digital archive of all electronic publications. (Note that *Plant Methods *will not levy additional page or colour charges and any number of colour figures and photographs can be included, at no extra cost). There will be no APCs for the first 6 months after launch, and after that time those authors whose institutions subscribe to BioMed Central will still be able to publish free of charge. In cases of lack of funds, authors' requests for waivers, if made at the time of submission, will be considered on a case-by-case basis.

On behalf of the Editorial Board, we hope you will support us in our efforts to make *Plant Methods *a success. We very much look forward to receiving your submissions.

## Competing interests

The majority of the APC is used by BioMed Central to fund the web services that host the journal and the submission and review system However, a small proportion of the APC is returned to the editorial office to support running costs associated with the journal.
